# High Seropositivity to *Coxiella burnetii* Among Goats Sampled in Southwestern Jeonnam Province, South Korea: A Pilot Study

**DOI:** 10.3390/ani16172797

**Published:** 2026-09-05

**Authors:** Eun-Yeong Bok, Chae-Rim Kwak, Han Gyu Lee, Duhyun Kim, Eun-Do Lee, Kwan-Woo Kim, Ara Cho, Younghun Jung, Seog-Jin Kang, Bock-Gie Jung

**Affiliations:** 1 Animal Genetic Resources Research Center, National Institute of Animal Science, Rural Development Administration, Hamyang 50000, Republic of Korea; eybok@korea.kr (E.-Y.B.); led9126@korea.kr (E.-D.L.); bgring@korea.kr (K.-W.K.); 2 Department of Veterinary Microbiology, College of Veterinary Medicine, Chonnam National University, Jeonnam-Gwangju Special Metropolitan City 61186, Republic of Korea; cofladb71@gmail.com (C.-R.K.); pepe0102@naver.com (D.K.); 3 Division of Animal Diseases & Health, National Institute of Animal Science, Rural Development Administration, Wanju 55365, Republic of Korea; gyu92@korea.kr (H.G.L.); aracho85@korea.kr (A.C.); vet9973@korea.kr (Y.J.); hijin@korea.kr (S.-J.K.)

**Keywords:** *Coxiella burnetii*, Q fever, zoonoses, seropositivity, goats

## Abstract

*Coxiella burnetii* is the causative agent of Q fever, a zoonotic disease of public health concern. Despite the recent expansion of the domestic goat industry in Korea, epidemiological data regarding *C. burnetii* exposure in Korean goat populations remain limited. This pilot study aimed to assess the serological distribution of *C. burnetii* exposure in the southwestern region of Jeonnam Province, the major goat-breeding region of Korea. Goat serum samples were originally collected for routine Foot-and-Mouth Disease monitoring in 2025. Using a two-stage random sampling approach, 92 samples were selected from 16 farms across five southwestern counties of Jeonnam Province. Specific antibodies against *C. burnetii* were detected using a commercial ELISA. The overall unadjusted apparent seropositivity was 44.6% (41/92). By county, seropositivity was 61.1% in both Haenam and Wando, 38.9% in Gangjin, 36.8% in Yeongam, and 26.3% in Jangheung. Furthermore, the overall herd-level apparent seropositivity was 68.8% (11/16). At the herd level within the surveyed farms, intra-herd seropositivity ranged from 0.0% to 100.0%, showing wide variation among the tested farms. The results indicate high serological exposure to *C. burnetii* within the surveyed southwestern counties. While constrained by a small sample size, these findings show that targeted longitudinal surveillance is required in this region. Expanding systematic monitoring efforts nationwide is recommended to establish effective veterinary and public health control measures.

## Simple Summary

Q fever is a zoonotic disease that can spread from infected animals—frequently goats, sheep, and cattle—to humans, posing a significant public health risk. Recently, goat farming has expanded in South Korea, with Jeonnam Province being the largest producing region. However, information regarding Q fever exposure in these goat populations remains highly limited. To address this gap, we tested goat blood samples from 16 farms across five counties in southwestern Jeonnam Province for antibodies against *Coxiella burnetii*, the bacteria responsible for Q fever. We discovered a high apparent seropositivity, with 44.6% of the tested goats showing positive results. This indicates substantial historical or recent exposure to the bacteria in this major farming region, rather than definitively confirming active infection. Because animals actively shedding the bacteria can transmit the disease to humans—putting farmers, veterinarians, and slaughterhouse workers at serious risk—these findings highlight a clear occupational and public health hazard. We strongly recommend implementing continuous, nationwide disease monitoring programs incorporating molecular diagnostics to better understand the true transmission dynamics and to protect both animal welfare and human health.

## 1. Introduction

*Coxiella burnetii* is an obligate intracellular bacterium and the causative agent of Q fever, a zoonosis affecting both veterinary and public health [1]. Although its broad host range complicates its epidemiology, domestic ruminants—particularly goats, sheep, and cattle—are recognized as the primary reservoirs [2]. In goats, the infection is frequently associated with severe reproductive disorders, including abortion, stillbirth, and endometritis, and the delivery of weak kids, which collectively contribute to elevated neonatal mortality and substantial economic losses [3]. In humans, transmission usually occurs through the inhalation of contaminated aerosols, resulting in clinical manifestations that range from mild flu-like symptoms to severe conditions like endocarditis [4].

In South Korea, the demand for goat meat and extracts has grown recently because these products are widely perceived as health supplements [5,6]. Consequently, the domestic goat population has steadily expanded, reaching 618,507 heads nationwide by 2026 [7]. Jeonnam Province is the primary goat-producing region, accounting for approximately 20% (123,930 heads) of the national herd [7]. Despite being managed as a statutory notifiable disease in both the veterinary and human health sectors in Korea due to the zoonotic risk of *C. burnetii* [8], comprehensive epidemiological data remain scarce. While previous Korean goat seroprevalence studies have been geographically limited to other provinces such as Jeonbuk [9] and Gyeongbuk [10], and broader national surveys have primarily focused on other livestock species [11,12,13], specific and intensive epidemiological data focusing on the southwestern region of Jeonnam Province—the most concentrated goat-producing area—were unavailable.

The lack of surveillance data in the largest production area leaves the national disease monitoring framework incomplete. Evaluating the serological status in this region is necessary to assess the risk to local agriculture and to understand the potential for zoonotic transmission. Therefore, this pilot study aimed to determine the serological distribution of *C. burnetii* exposure in the southwestern region of Jeonnam Province, the major goat-breeding area of South Korea.

## 2. Materials and Methods

### 2.1. Ethics Statements

This study was reviewed and approved by the Institutional Animal Care and Use Committee (IACUC) of Chonnam National University (Approval No. CNU IACUC-YB-2026-74). As the research relied on archived residual serum samples originally collected for routine Foot-and-Mouth Disease (FMD) surveillance, no animals underwent additional handling or sampling. All samples were processed anonymously to maintain confidentiality.

### 2.2. Study Design and Sample Collection

**Figure 1:**
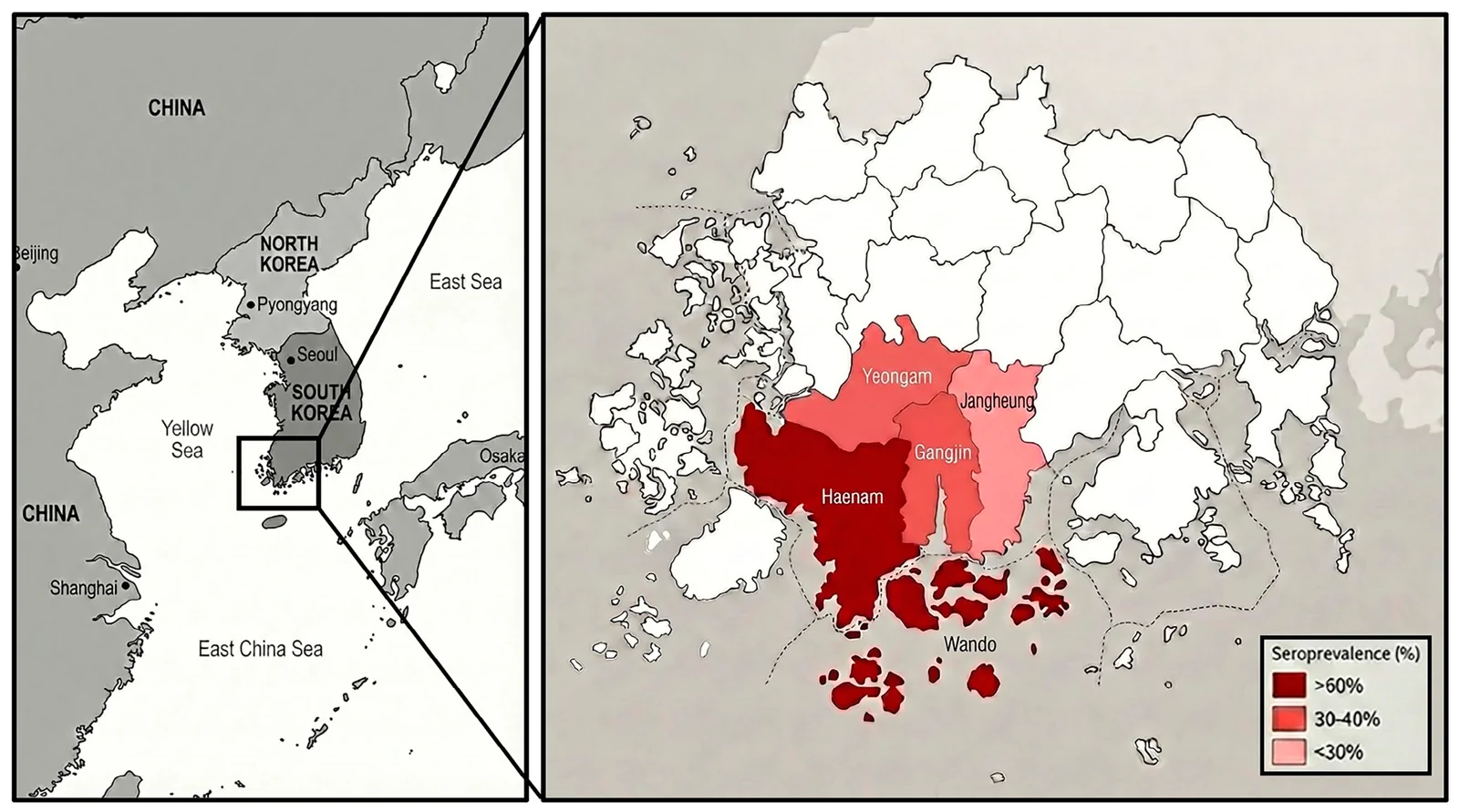
Geographic distribution of *Coxiella burnetii* seropositivity in goats across five counties in Jeonnam Province, South Korea. Data are based on serum samples collected in 2025. Color intensity indicates apparent seropositive rates: dark red for high rates (>60%; Haenam [61.1%] and Wando [61.1%]); medium red for moderate rates (30–40%; Gangjin [38.9%] and Yeongam [36.8%]); and light red for the lowest rate (<30%; Jangheung [26.3%]). High seropositivity was primarily concentrated in the southwestern coastal and island regions. The dashed lines indicate city and county boundaries.

This study was conducted in the southwestern region of Jeonnam Province, the largest goat-producing region in the Republic of Korea. In 2026, we utilized archived residual serum samples originally collected in 2025 for the national FMD surveillance program. Because the sampling frame was dictated by the availability of these residual sera, the sample size per farm (2 to 7 goats) was constrained by the remaining volume rather than a priori statistical power calculations for within-herd seroprevalence estimation. To minimize selection bias within this available sample pool, a two-stage random sampling approach was applied: 16 farms were randomly selected across five southwestern counties (Haenam, Wando, Gangjin, Yeongam, and Jangheung), and then 92 goat serum samples were randomly sub-sampled from these herds (Figure 1). Ultimately, this strategy provided valuable seroepidemiological data without requiring further animal sampling.

### 2.3. Serological Analysis

The archived residual sera used in this study were originally collected for routine FMD monitoring. Following the initial FMD serological testing, the remaining serum aliquots were immediately frozen and stored strictly at −80 °C. To ensure antibody stability and preserve the integrity of the samples, all archived sera were subjected to only a single freeze–thaw cycle prior to the *C. burnetii* enzyme-linked immunosorbent assay (ELISA) analysis. Antibodies against *C. burnetii* were detected using a commercial indirect ELISA kit (ID Screen^®^ Q Fever Indirect Multi-species, Lot No. P38, IDvet, Grabels, France) according to the manufacturer’s instructions. For the assay run, the internal positive and negative controls strictly met the manufacturer’s acceptance criteria, ensuring the validity of all test plates. Briefly, serum samples were diluted and incubated in antigen-coated wells. Optical density (OD) was measured at 450 nm using a microplate reader. The sample-to-positive (S/P) percentage was calculated using the following formula: S/P% = ((OD_sample_ − OD_NC_)/(OD_PC_ − OD_NC_)) × 100, where NC and PC represent the mean optical densities of the negative and positive controls, respectively. Samples were interpreted based on the S/P% (sample-to-positive) ratio cut-offs specified in the manual (positive if S/P% ≥ 50%, negative if S/P% ≤ 40%, and doubtful if 40% < S/P% < 50%). To prevent overestimating seropositivity, doubtful samples were conservatively classified as negative.

### 2.4. Statistical Analysis

Seropositivity was calculated as the proportion of positive samples among the total tested. As a preliminary investigation, data were analyzed using descriptive statistics to report the distribution of positive cases. The 95% confidence intervals (CIs) for the seropositivity estimates were calculated based on the Wilson score interval method using GraphPad Prism software version 11.0.2 (GraphPad Software, Boston, MA, USA).

## 3. Results

### 3.1. Seropositivity of C. burnetii in Goats by Region

In this study, a total of 92 goat serum samples from 16 farms across five counties in the southwestern region of Jeonnam Province were tested. According to the manufacturer’s threshold criteria, 2 out of 92 samples yielded doubtful results. To maintain high diagnostic specificity and avoid false-positive classifications, these doubtful samples were conservatively categorized as negative in our final seropositivity calculations. Consequently, the overall apparent seropositivity for *C. burnetii* was 44.6% (41/92; 95% CI, 34.8–54.7). Geographically, the seropositivity varied among the surveyed counties. The highest rates occurred in the coastal and island counties of Haenam (61.1%; 11/18) and Wando (61.1%; 11/18). Rates were lower in Gangjin (38.9%; 7/18) and Yeongam (36.8%; 7/19), and Jangheung had the lowest rate at 26.3% (5/19). Figure 1 shows the spatial distribution of these positive cases within the southwestern part of the province.

### 3.2. Farm-Level Heterogeneity in Seropositivity

The overall herd-level apparent seropositivity was 68.8% (11/16), indicating that 11 out of the 16 surveyed farms housed at least one seropositive goat. At the herd level within the surveyed counties, intra-herd seropositivity rates ranged from 0.0% to 100.0% (Table 1). Both farms with no positive animals (0.0%) and highly positive (100.0%) farms were located within the same administrative districts, such as Yeongam and Haenam. In contrast, all tested farms in Wando had at least one seropositive animal.

## 4. Discussion

The overall apparent seropositivity of 44.6% observed among the tested goat samples is substantially higher than the rates previously reported for other domestic livestock species in Korea. Specifically, earlier national surveys reported seroprevalence rates of 9.5% to 11.6% in cattle [13], 6.8% in pigs [11], and 1.3% in horses [12]. Furthermore, this figure exceeds the seroprevalence frequently observed in goat populations both domestically—estimated at 15.0% to 19.0% [14]—and internationally, including regions such as Costa Rica (1.8%) [15], Laos (4.1%) [16], Nigeria (12.0%) [17], and Egypt (28.2%) [18]. Jeonnam Province accounts for the largest share of goat production in South Korea. Although this pilot study did not cover the entire province, the findings from the five southwestern counties provide clear serological evidence of substantial *C. burnetii* exposure within this major production area.

While the descriptive data showed numerical variations in seropositivity among the five surveyed counties (ranging from 26.3% to 61.1%), attributing these differences to specific geographic or climatic factors is unwarranted. The surveyed counties are geographically adjacent and share similar environmental conditions. Thus, rather than indicating distinct regional risk factors, the detection of antibodies against *C. burnetii* across all these contiguous districts demonstrates that serological exposure is widely established throughout the surveyed southwestern area.

Similarly, the wide variation observed at the herd level—ranging from 0.0% to 100.0%—should be interpreted with caution. Because the sampling frame was based on available residual sera, resulting in a small number of tested animals per farm (2 to 7 goats), this sample size is statistically insufficient to robustly estimate true within-herd seroprevalence. Therefore, these extreme fluctuations likely reflect the statistical variability associated with small intra-herd sample sizes rather than absolute differences in farm-level biosecurity measures.

Because *C. burnetii* is a potent zoonotic pathogen, this widespread exposure in caprine reservoirs poses a direct threat to public health. Domestic ruminants serve as primary reservoirs and shed the organism heavily during parturition, creating a significant occupational hazard for local farmers, clinical veterinarians, and slaughterhouse workers. This risk is clearly reflected in human seroprevalence, which often correlates closely with livestock seropositivity. For instance, occupational cohorts in high-risk areas frequently show elevated seropositivity, such as 25.7% among small ruminant breeders in Egypt [18] and 24.1% among farm workers in Ecuador [19]. In Korea, massive human Q fever outbreaks have already been directly linked to infected goat farms [14]. The lack of overt clinical signs in seropositive animals further emphasizes the need to recognize this baseline exposure to prevent unrecognized zoonotic transmission. Ultimately, the serological evidence of widespread *C. burnetii* exposure within this major production area shows the critical need for integrated One Health surveillance.

This preliminary survey has certain methodological limitations. The primary constraint is the reliance on a limited number of archived residual sera, which restricts the statistical power required for comprehensive region-to-region or farm-to-farm comparisons. Consequently, the observed apparent seropositivity (44.6%) from these five southwestern counties cannot be directly generalized as the true population prevalence for the entire province or country but should be strictly interpreted as a regional epidemiological snapshot of the surveyed herds. Furthermore, antibody detection via ELISA confirms only prior or recent exposure to *C. burnetii*, not active infection or current bacterial shedding. Therefore, the reported seropositivity reflects historical exposure rather than active transmission dynamics. This highlights the absence of PCR testing as a significant limitation, since serology alone cannot definitively identify ongoing infections. Additionally, our conservative methodological approach of classifying doubtful ELISA results as negative serves as a minor limitation. While this strict threshold ensures high diagnostic specificity and prevents the overestimation of *C. burnetii* exposure, it may also result in a slight underestimation of the true seroprevalence. Nevertheless, by establishing serological evidence of high exposure in these core-producing counties, this study provides an essential epidemiological baseline.

To build upon this foundation and comprehensively understand the transmission dynamics of *C. burnetii*, future research requires more robust, multidisciplinary approaches. Recent epidemiological studies emphasize that herd-level risk factors, such as large herd size, high stocking density, and inadequate biosecurity practices during abortion events, play a critical role in the transmission dynamics of caprine coxiellosis [20,21]. Future large-scale longitudinal studies should integrate molecular diagnostics, such as PCR, to identify active bacterial shedding. In fact, recent molecular investigations in small ruminants highlight that concurrent PCR testing is essential, as seropositivity does not always correlate perfectly with actual environmental shedding and transmission risk [22]. Furthermore, studies tracking vaccinated and naturally infected goat herds have demonstrated prolonged environmental contamination, particularly in milking parlors, underscoring the persistence of molecular shedding [23]. Given the severe zoonotic potential of Q fever and its recent increasing trend in Korea [8], synchronizing veterinary monitoring with human occupational surveillance is essential. Establishing an effective One Health control strategy—integrating human, animal, and environmental domains—is strongly advocated by recent global surveillance reviews and the literature to combat this neglected zoonotic disease [8,24,25].

## 5. Conclusions

In conclusion, this pilot serological survey demonstrates a high apparent seropositivity for *C. burnetii* among sampled goats in southwestern Jeonnam Province, indicating substantial historical or recent exposure. However, because this survey relied on a limited number of archived samples and lacked molecular diagnostic tools, these findings cannot be used to definitively determine active bacterial transmission or the absolute regional disease burden. To establish a comprehensive epidemiological understanding, larger representative studies combining both serology and molecular detection (e.g., PCR) are strictly required.

## Figures and Tables

**Table 1:** Seropositivity for *Coxiella burnetii* in goats among 16 farms across five counties in Jeonnam Province, South Korea.

Region	Farm ID	No. Tested (*n*)	No. Positive (*n*)	Apparent Seropositivity (%)	95% CI *
Yeongam	A	6	0	0.0	0.0–39.0
	B	7	7	100.0	64.6–100.0
	C	6	0	0.0	0.0–39.0
	Subtotal	19	7	36.8	19.1–59.0
Haenam	A	7	5	71.4	35.9–91.8
	B	4	0	0.0	0.0–49.0
	C	7	6	85.7	48.7–97.4
	Subtotal	18	11	61.1	38.6–79.7
Wando	A	6	3	50.0	18.8–81.2
	B	6	5	83.3	43.6–97.0
	C	6	3	50.0	18.8–81.2
	Subtotal	18	11	61.1	38.6–79.7
Gangjin	A	2	0	0.0	0.0–65.8
	B	5	3	60.0	23.1–88.2
	C	4	2	50.0	15.0–85.0
	D	7	2	28.6	8.2–64.1
	Subtotal	18	7	38.9	20.3–61.4
Jangheung	A	6	3	50.0	18.8–81.2
	B	6	0	0.0	0.0–39.0
	C	7	2	28.6	8.2–64.1
	Subtotal	19	5	26.3	11.8–48.8
Total		92	41	44.6	34.8–54.7

* CI, confidence interval (calculated using the Wilson score interval method).

## Data Availability

The datasets used and/or analysed during the current study are available from the corresponding author upon reasonable request.
